# Supplementary material for: Parechovirus infection in human brain organoids: host innate inflammatory response and not neuro-infectivity correlates to neurologic disease
Source: Nat Commun. 2024 Mar 21;15:2532. doi: 10.1038/s41467-024-46634-9 (PMC10958052; doi:10.1038/s41467-024-46634-9)
Supplement: Supplementary file 1 — Supplementary Information [file 41467_2024_46634_MOESM1_ESM.pdf]

Supplementary material

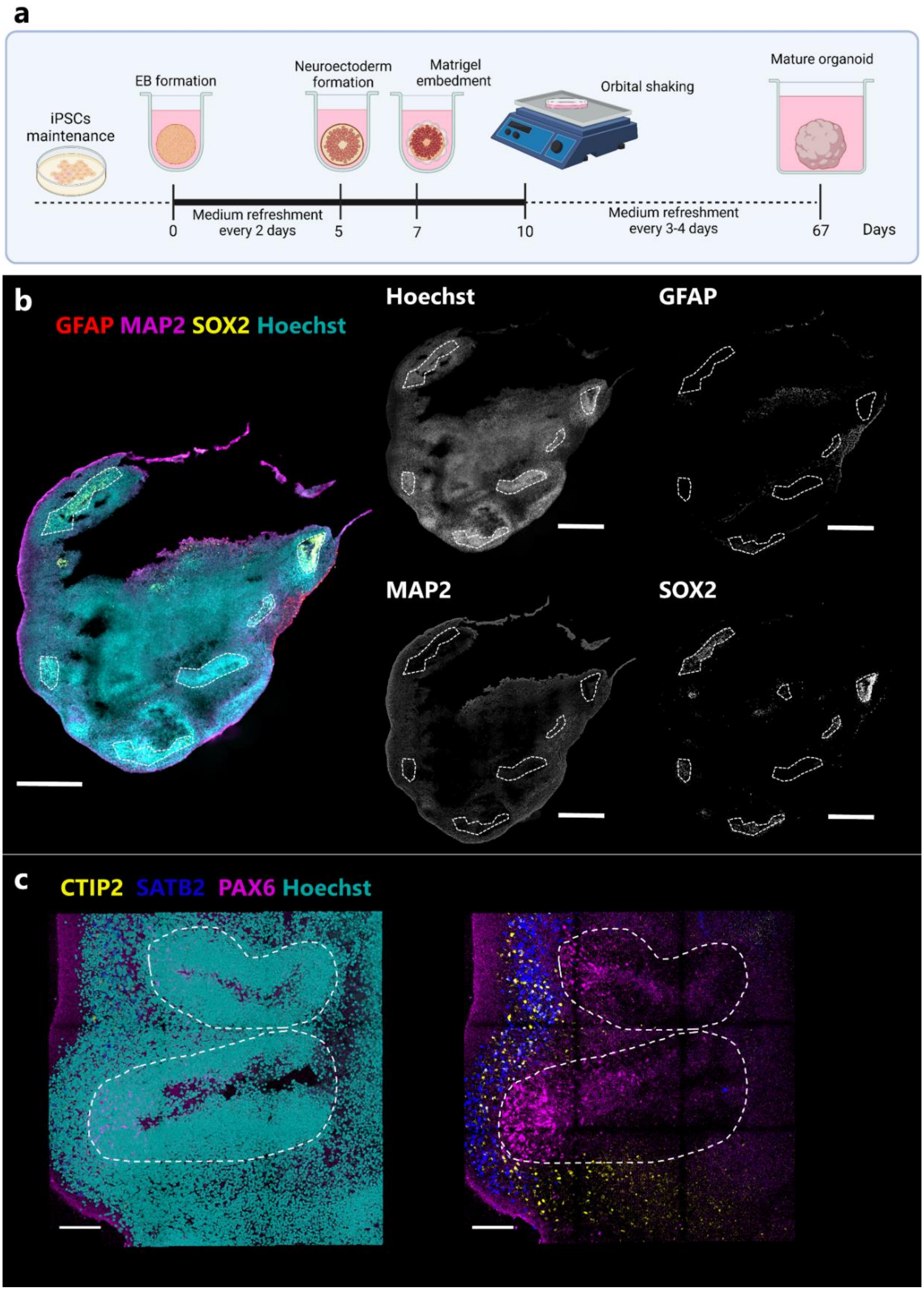

Supplementary Figure 1. Characterization of unguided neural organoids (UNO) by immunofluorescence staining. a, Schematic representation of the protocol followed to obtain UNOs. **b**, Representative image of an UNO section with the expected cytoarchitecture. Astrocyte-rich areas (GFAP, red) and neuron-rich areas (MAP2, magenta) are located outside the ventricular-like zones (VZs) that are high in neural progenitor cells (SOX2, yellow). Dashed lines are marked around VZs. Scale bar 500 µm. **c**, Representative image of cortical neuron layering in UNOs. PAX6 staining (magenta) show neural progenitor cells in the VZs surrounded by early born deep-layer neurons (CTIP2, yellow), and late-born superficial layer neurons (SATB2, blue). Scale bar 100µm. In both cases nuclei were stained with Hoechst (cyan).

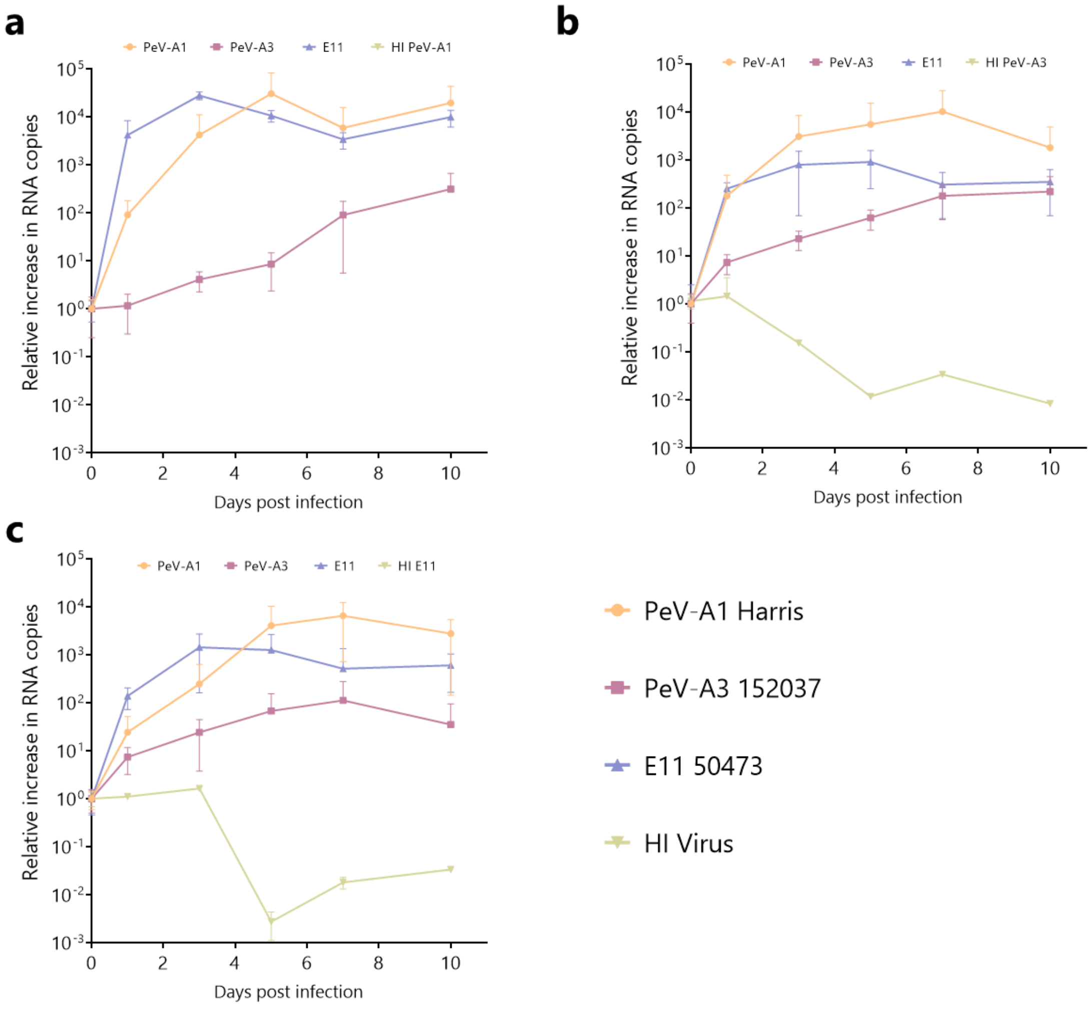

Supplementary Figure 2. a, Relative increase in viral RNA copies in samples collected from supernatant at different time points for PeV-A1, PeV-A3, E11, heat-inactivated PeV-A1, **b**, heat-inactivated PeV-A3, **c**, and heat-inactivated E11 on UNOs. In all cases, data represents the mean ± SEM of three technical replicates in one batch of organoids. HI = heat inactivated. HI PeV-A1 RNA copies were undetectable. Source data are provided as a Source Data file.

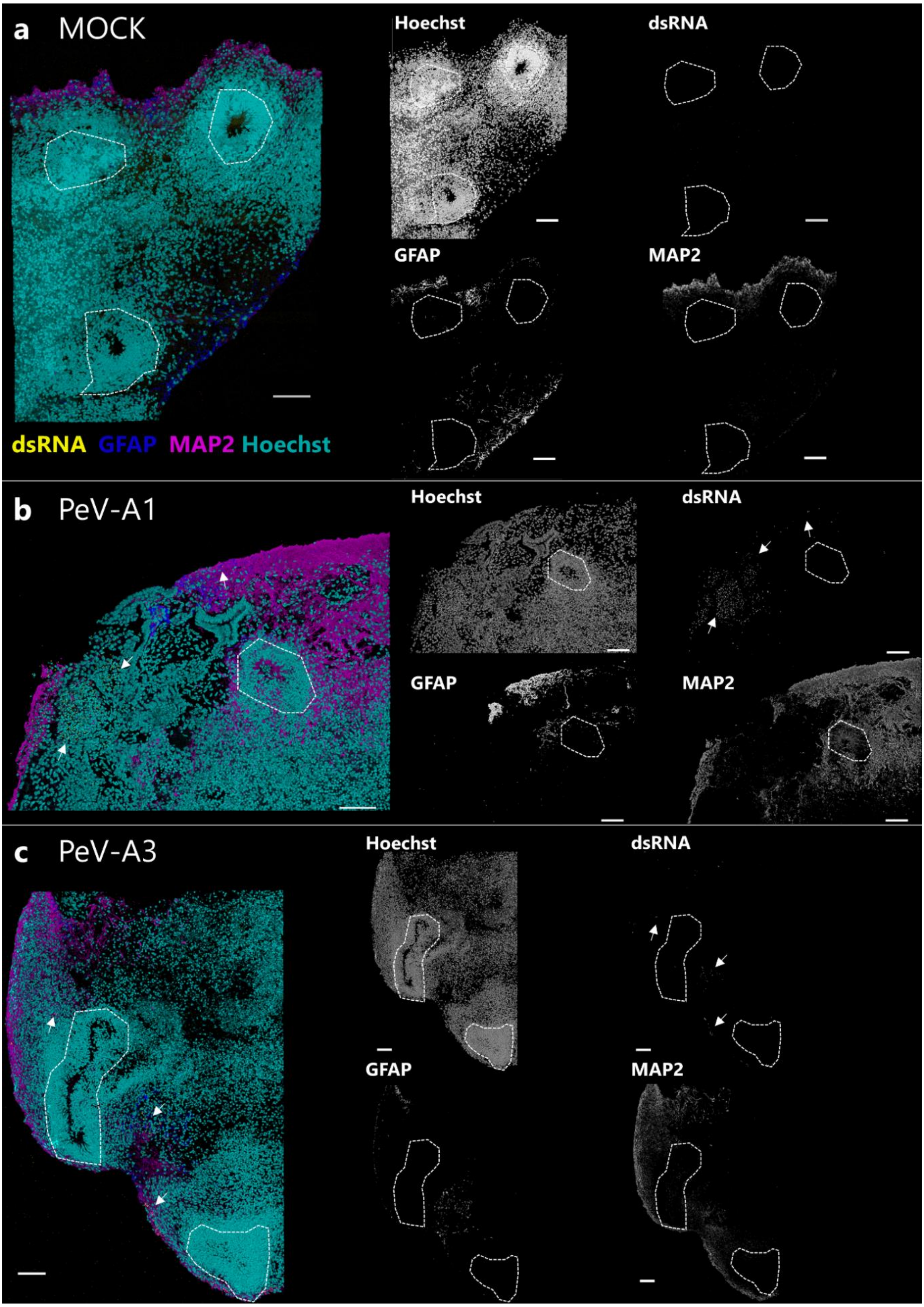

Supplementary Figure 3. Representative confocal z-stacks from **a,** MOCK, **b,** PeV-A1, and **c,** PeV-A3 infected UNOs at 10 dpi. Stained for nuclei (cyan), and immunolabelled for dsRNA (yellow), astrocytes (GFAP, blue) and neurons (MAP2,magenta). Dashed lines are around VZs. Scale bar 100 µm. Arrows indicate regions with positive dsRNA staining.

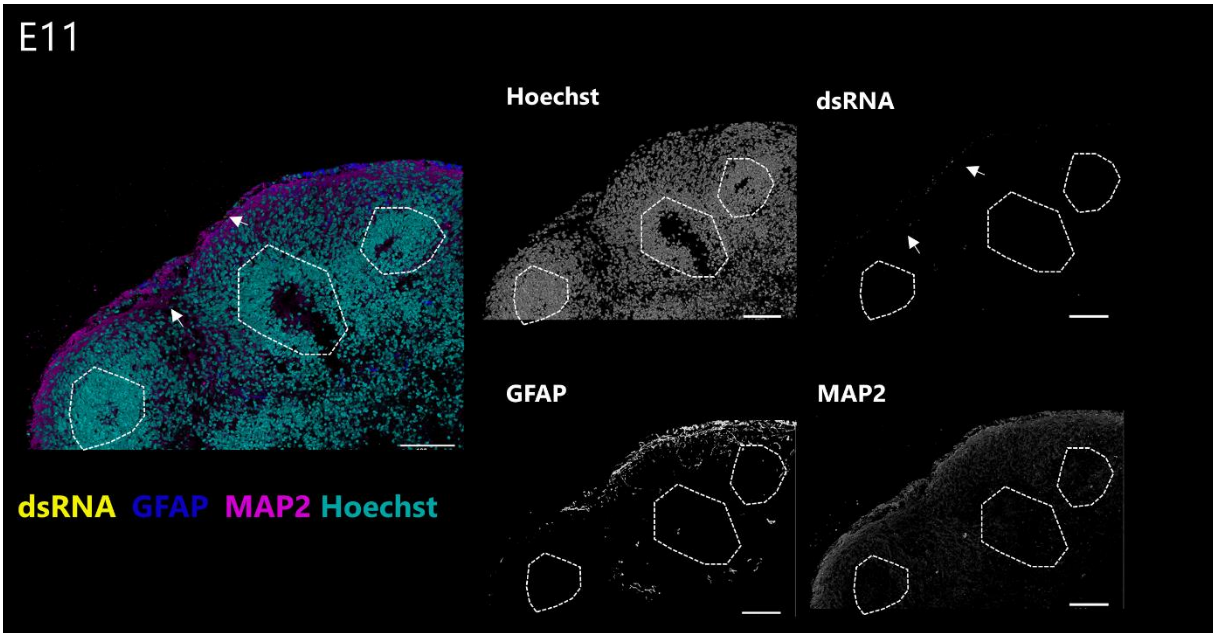

Supplementary Figure 4. Confocal z-stacks from E11 infected UNOs at 10 dpi. Labelled for nuclei (cyan), dsRNA (yellow), GFAP (blue) and MAP2 (magenta). Arrows indicate regions with positive dsRNA staining.

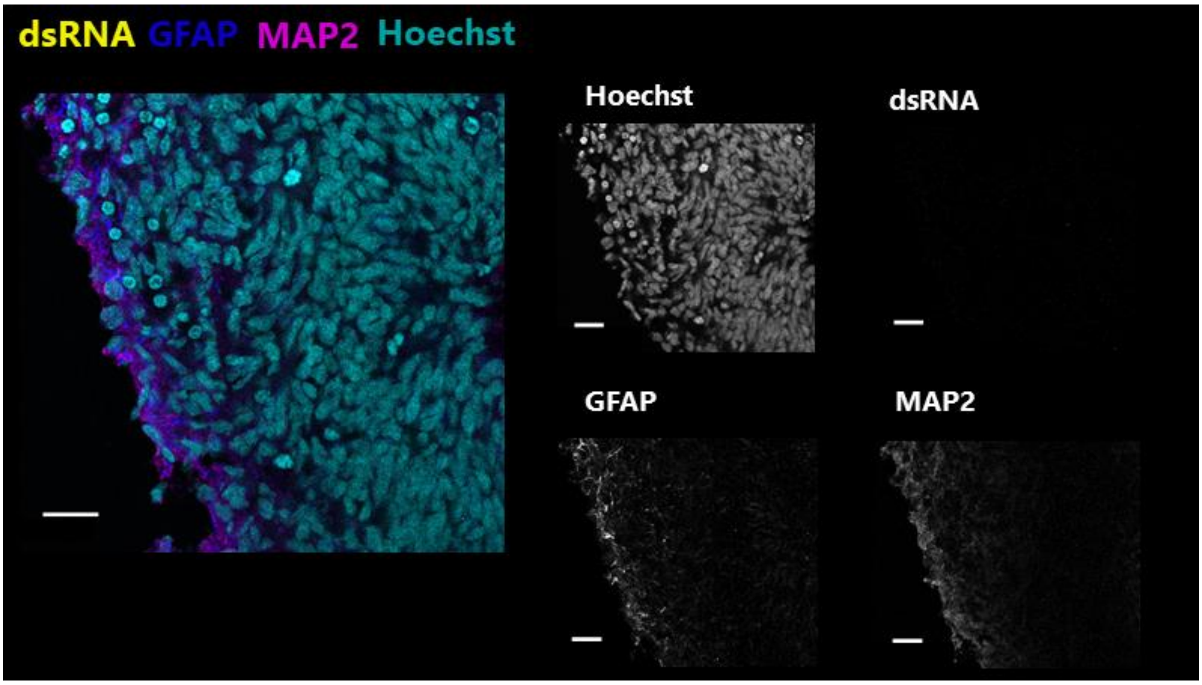

Supplementary Figure 5. Confocal image of MOCK infected UNOs. Labelled for nuclei (cyan), and immunolabelled for dsRNA (yellow), GFAP (blue), and MAP2 (magenta). Scale bars 20 µm.

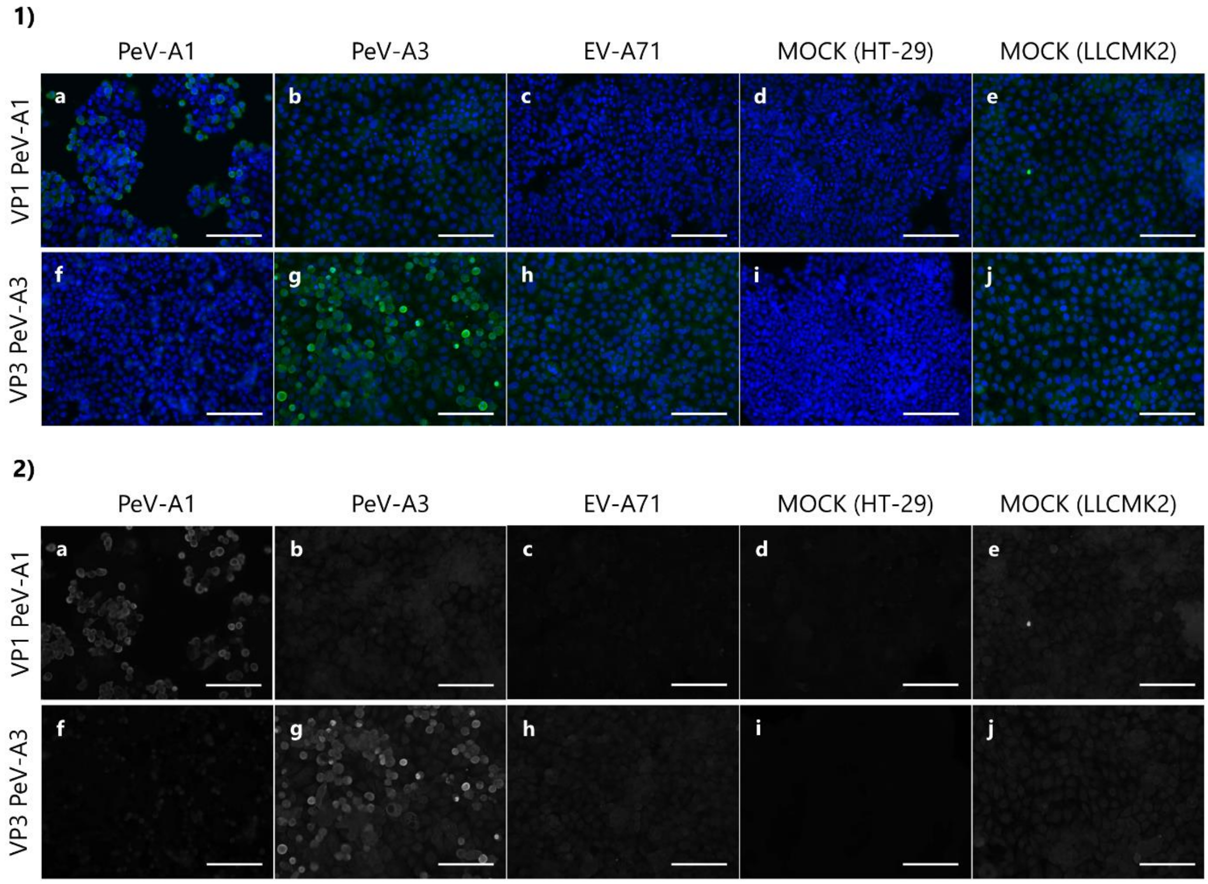

Supplementary Figure 6. VP1/VP3 specific antibodies validation. Representative fluorescence microscopy images of HT-29 (a, c, d, f, h, i) or LLMCK2 (b, e, g, j) cells infected with PeV-A1 Harris (a, f), PeV-A3 152037 (b, g), EV-A71 (c, h), or uninfected (d, e, i, j). In panel 1 cells were stained for nuclei (blue) and PeV-A1 VP1 (a-e) or PeV-A3 VP3 (f-j) (green). Panel 2 represents gray scale images of the VP1/VP3 stainings representing only the green channel. VP specific antibodies were used at 1:300 dilution to allow for the best signal-to-noise ratio (data not shown). Scale bar 125 µm.

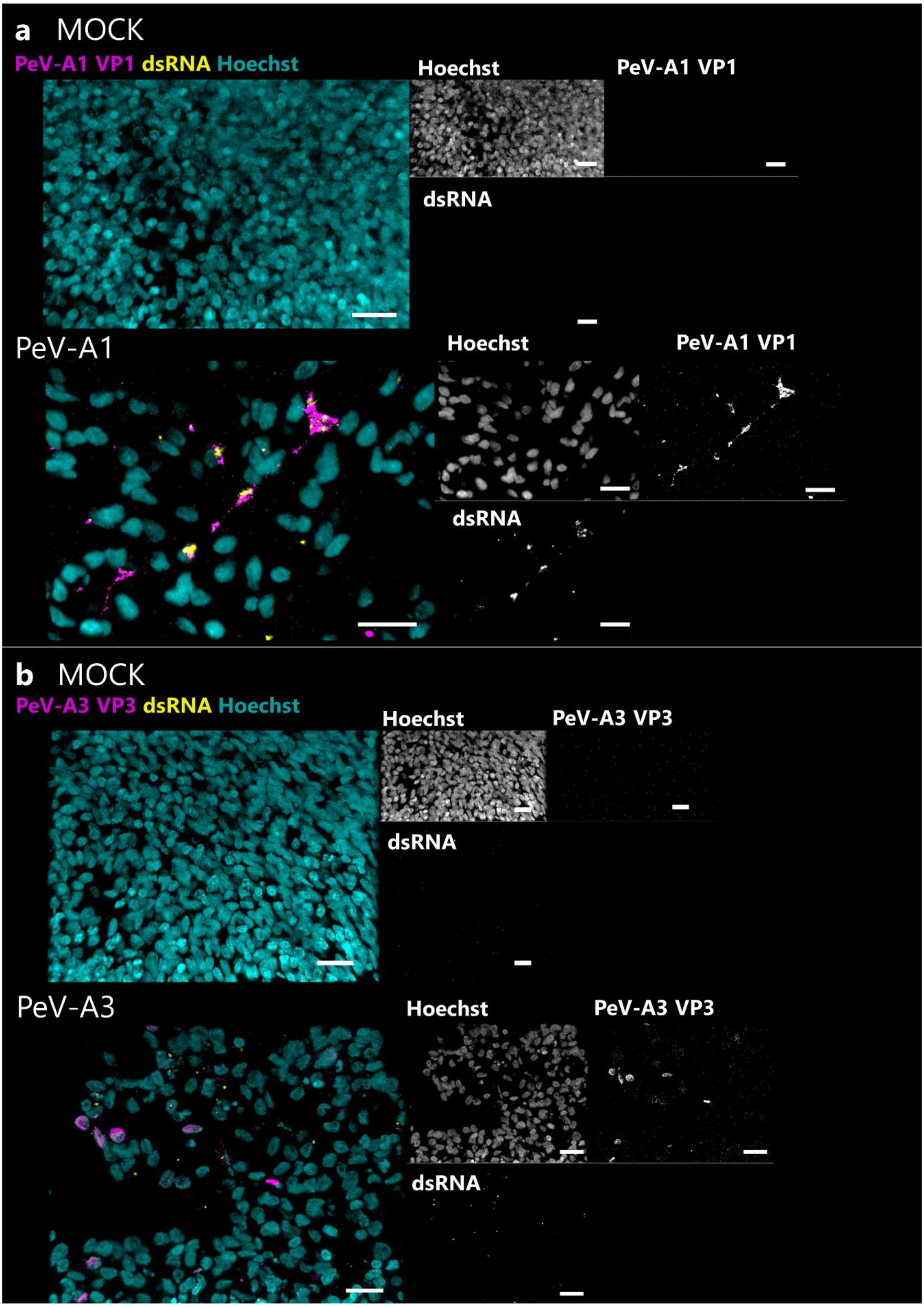

Supplementary Figure 7. Representative confocal images from **a**, MOCK and PeV-A1 infected UNOs and stained for nuclei (cyan), and immunolabelled for PeV-A1 VP1 (magenta) and dsRNA (yellow). **b**, MOCK and PeV-A3 infected UNOs and stained for nuclei (cyan), and immunolabelled for PeV-A3 VP3 (magenta) and dsRNA (yellow). Scale bar 20 µm.

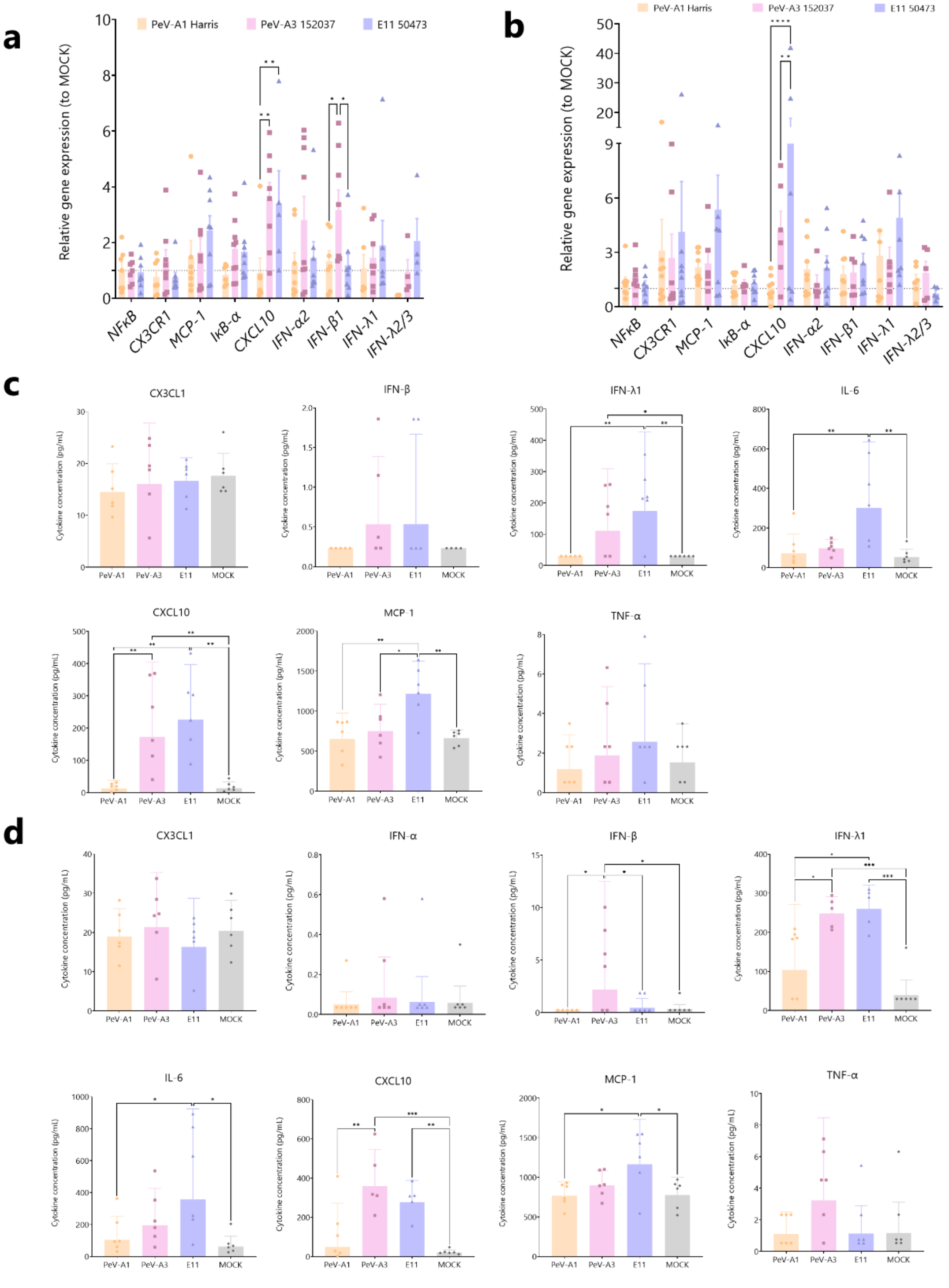

Supplementary Figure 8. PeV-A3 leads to upregulation of the inflammatory response. a, Quantification of relative expression of cytokines to MOCK infected organoids at **a,** 5 dpi and **b,** 10 dpi by RT-qPCR. All data corresponds to the geometric mean ± geometric SD of three technical replicates for three batches of organoids. Values above the dashed line represent an upregulation of the gene expression relative to the MOCK. Statistical significance was analysed by two-way ANOVA with Tukey's multiple comparisons tests for each specific cytokine. **c-d** Quantification of cytokines by Luminex present in supernatant of UNOs infected with PeV-A1, PeV-A3, E11, or MOCK infected at **c,** 5 dpi and **d,** 10 dpi. For **c,** cytokines for which most values were below the LOD were removed. All data corresponds to the geometric mean ± geometric SD of two technical replicates (individual organoids) in three batches of organoids (individual experiments). Statistical significance was assessed using a One-way ANOVA with Tukey's multiple comparisons for each cytokine individually, * *P*-value < 0.05; ** *P*-value <0.01; *** *P*-value <0.001; **** *P*-value <0.0001. Source data are provided as a Source Data file.

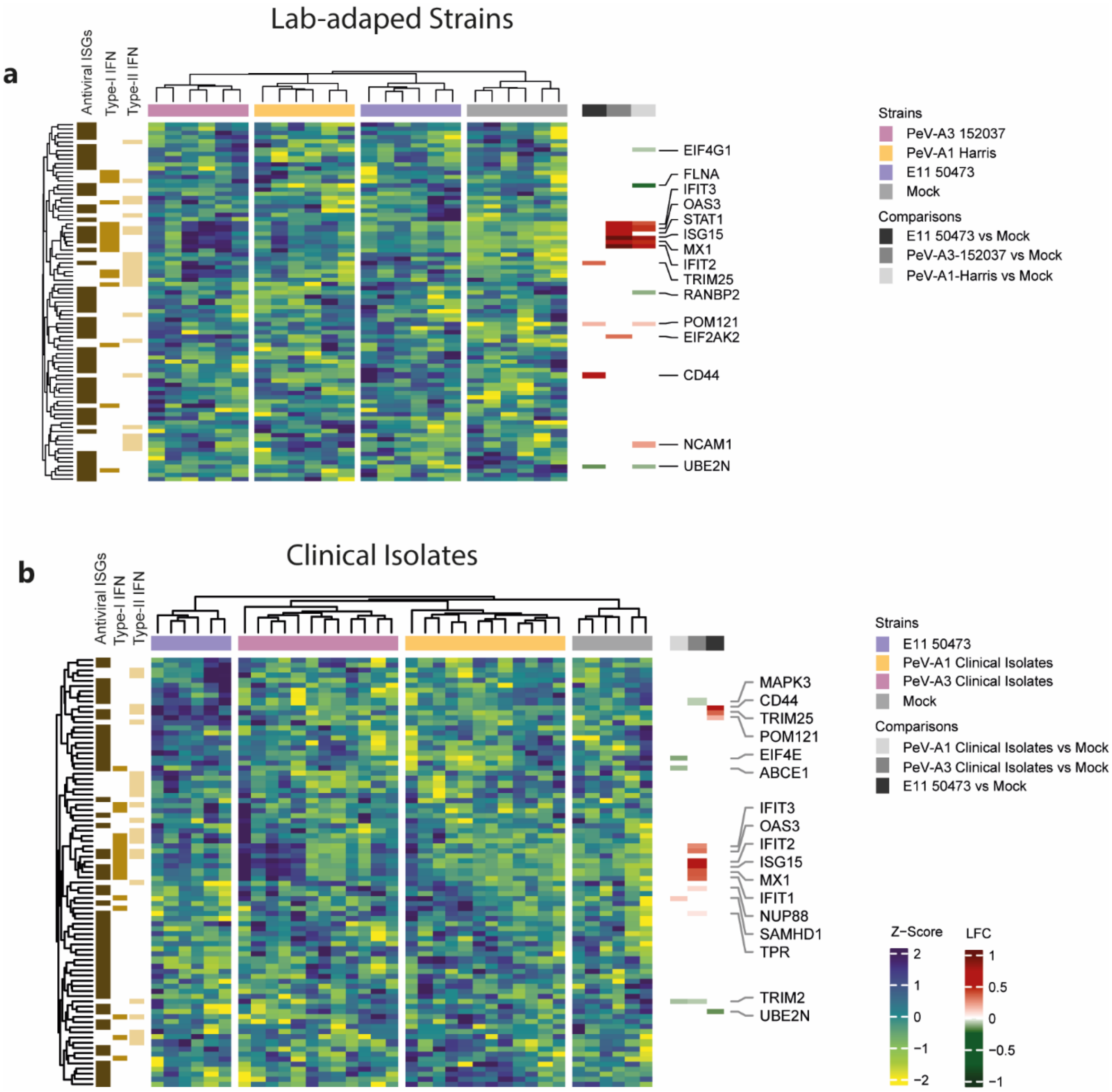

Supplementary Figure 9. Hierarchical clustering of DAPs involved in the regulation of interferon pathways. ISG and IFN-related DAPs upregulated in UNOs upon infection with PeV-A1, PeV-A3 and E11 using either **a,** Lab-adapted strains of PeV-A. **b,** Clinical isolates. The four columns in the heatmap represent protein expression levels in each sample of PeV-A1, PeV-A3, E11 and Mock infected UNOs. Z-scores represented by purple and yellow indicate the higher and lower expression levels, respectively. The colums on the right represent the significantly DAPs when normalized to the Mock. Up and downregulation of genes is indicated by green or red respectively. DAPs, differentially expressed proteins. LFC, log-fold change. Source data are provided as a Source Data file.

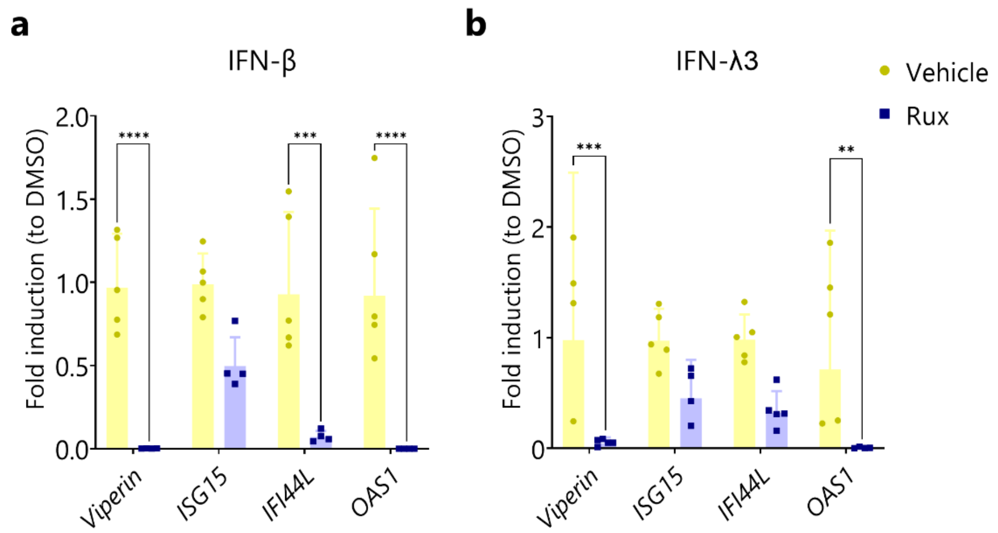

Supplementary Figure 10. Rux inhibition of IFN-stimulated ISG induction in UNOs. RT-qPCR analysis of ISGs upon stimulation with **a**, 500 ng of IFN-β, or **b**, 500 ng of IFN-λ3 and treated with Rux or vehicle control (DMSO). Data corresponds to the geometric mean fold induction from vehicle treated organoids ± geometric SD of five different organoids. Statistical significance was analyzed by two-way ANOVA with Šídák's multiple comparisons tests for each specific cytokine, ** *P*-value <0.01; *** *P*-value <0.001; **** *P*-value <0.0001. Source data are provided as a Source Data file.

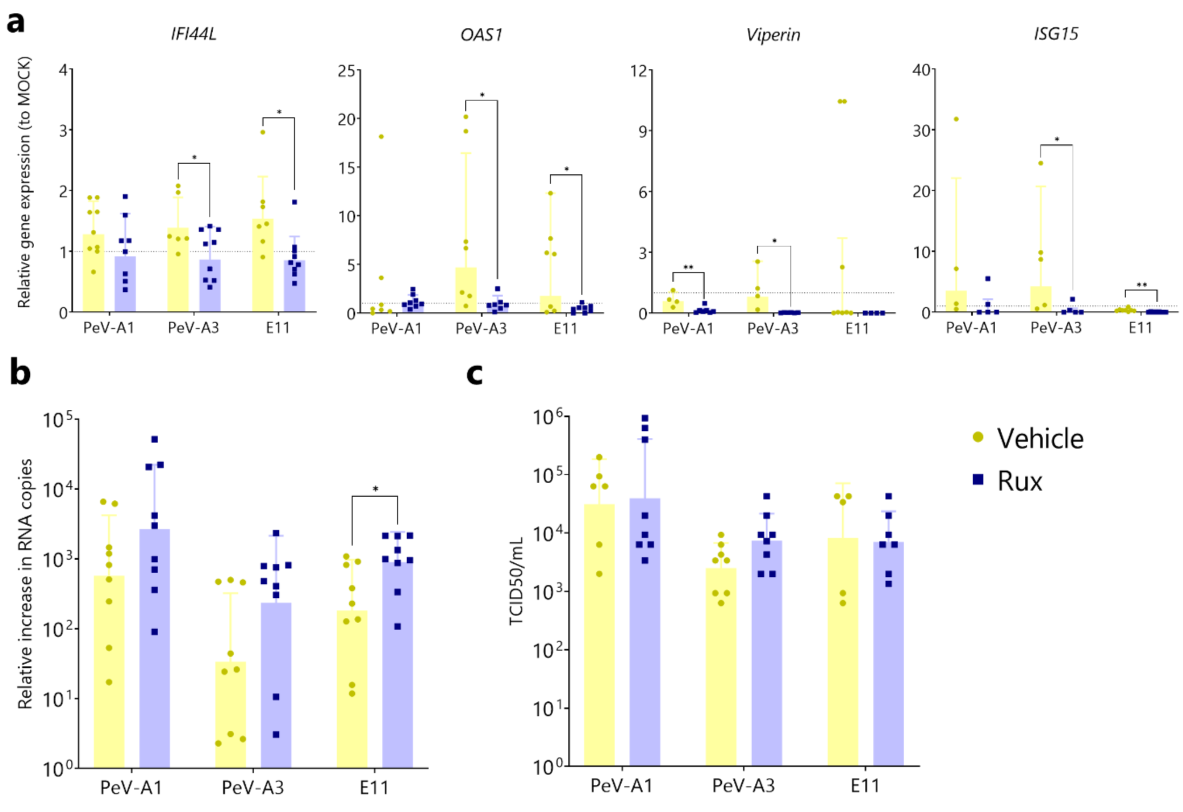

Supplementary Figure 11. **a**, Relative gene expression of ISGs in Rux or vehicle (DMSO) treated organoids. **b**, Relative increase in RNA copies at 10 dpi for Rux or vehicle treated organoids from supernatant samples. **c**, Virus titer at 10 dpi from supernatant collected samples of Rux or vehicle treated organoids. Titers were determined by TCID50. In all cases, data corresponds to the geometric mean ± geometric SD of three technical replicates (individual organoids) in three batches (individual experiments) of organoids. Statistical significance was determined using an unpaired two-tailed t-test, * *P*-value < 0.05. For **a** values above the dashed line represent an upregulation of the gene expression relative to the MOCK. Source data are provided as a Source Data file.

Supplementary Table 1. List of primers used in this study with forward and reverse sequences.

Target	Forward sequence (5'-3')	Reverse sequence (5'-3')
PeV-A	CTGGGGGCCAAAAGCCA	GGTACCTTCTGGGCATCCTTC
PeV-A Probe	(6'FAM)-AAACACTAGTTGTAWGGCCC	---
Pan-EV	GGCCCTGAATGCGGCTAAT	GGGATTGTCACCATAAGCC
CXCL10	GGTGAGAAGAGATGTCTGAATCC	GTCCATCCTTGGAAGCACTGCA
CX3CR1	GTGGTGCTGACAAAGCTTGGAA	TCACTGGGTGCCATCGTAAGAA
IFI44L	TGCACTGAGGCAGATGCTGCG	TCATTGCGGCACACCAGTACAG
IFN-α2	TGGGCTGTGATCTGCCTCAAAC	CAGCCTTTTGGAACTGGTTGCC
IFN-β1	CTTGGATTCCTACAAAGAAGCAGC	TCCTCCTTCTGGAACTGCTGCA
IFN-λ1	AACTGGGAAGGGCTGCCACATT	GGAAGACAGGAGAGCTGCAACT
IFN-λ2/3	TCGCTTCTGCTGAAGGACTGCA	CCTCCAGAACCTTCAGCGTCAG
IκB-α	GCTGAAGAAGGAGCGGCTACT	TCGTACTCCTCGTCTTTCATGGA
ISG15	CTCTGAGCATCCTGGTGAGGAA	AAGGTCAGCCAGAACAGGTCGT
MCP-1	CCTTCATTCCCCAAGGGCTC	GGTTTGCTTGTCCAGGTGGT
NFκB	GCAGCACTACTTCTTGACCACC	TCTGCTCCTGAGCATTGACGTC
OAS1	AGGAAAGGTGCTTCCGAGGTAG	GGACTGAGGAAGACAACCAGGT
RPLP0	TGGTCATCCAGCAGGTGTTCGA	ACAGACACTGGCAACATTGCGG
RPLP2	TCTTGGACAGCGTGGGTATCGA	CAGCAGGTACACTGGCAAGCTT
Viperin	CCAGTGCAACTACAAATGCGGC	CGGTCTTGAAGAAATGGCTCTCC

Supplementary Table 2. List of antibodies used in this study.

Antigen (abbreviation)	Host	Dilution factor	Company	Catalogue #
Special AT-rich sequence-binding protein 2 (SATB2)	Mouse	1:400	Abcam	Ab51502
Chicken ovalbumin upstream promoter transcription factor-interacting protein 1 (CTIP2)	Rat	1:500	Abcam	Ab18465
Paired box protein 6 (PAX6)	Rabbit	1:300	Stemcell Technologies	STC60094
Glial fibrillary acidic protein (GFAP)	Goat	1:500	Abcam	Ab53554
Sex determining region Y-box 2 (SOX2)	Rabbit	1:400	Cell Signaling Technologies	34516
Microtubule-associated protein 2 (MAP2)	Mouse	1:500	Thermo Fisher Scientific	MA5-12826
PeV-A1 VP1 LPAPKVTSSRALRGDMAN	Rabbit	1:300	Thermo Fisher Scientific	Custom made
PeV-A3 VP3 ERAFYDPRTAGSKSRFDD	Rabbit	1:300	Thermo Fisher Scientific	Custom made
dsRNA clone rJ2	Mouse	1:60	Sigma-Aldrich	MABE1134
**Secondary Antibody**	**Host**	**Dilution factor**	**Company**	**Catalogue #**
Anti-mouse Alexa Fluor 546	Donkey	1:750	Thermo Fisher Scientific	A10036
Anti-rat Alexa Fluor 488 (donkey)	Donkey	1:750	Thermo Fisher Scientific	A48268
Anti-rabbit Alexa Fluor 680 (donkey)	Donkey	1:750	Abcam	Ab175772
Anti-goat Alexa Fluor 594 (donkey)	Donkey	1:750	Thermo Fisher Scientific	A11058

Supplementary Table 3. Sample identities vs TMT channels employed.

Batch 1			Batch 2			Batch 3		
ID	Virus strain	TMT	ID	Virus strain	TMT	ID	Virus strain	TMT
1-1	PeV-A1 Harris	126	2-1	PeV-A1 Harris	126	3-1	PeV-A1 Harris	126
1-3	PeV-A1 Harris	131C	2-3	PeV-A1 Harris	131C	3-3	PeV-A1 Harris	131C
1-5	PeV-A1 52967	128N	2-5	PeV-A1 52967	128N	3-5	PeV-A1 52967	128N
1-6	PeV-A1 52967	134N	2-6	PeV-A1 52967	134N	3-6	PeV-A1 52967	134N
1-10	PeV-A1 51067	132N	2-10	PeV-A1 51067	132N	3-10	PeV-A1 51067	132N
1-12	PeV-A1 51067	130N	2-12	PeV-A1 51067	130N	3-12	PeV-A1 51067	130N
1-14	PeV-A3 152037	127N	2-14	PeV-A3 152037	127N	3-14	PeV-A3 152037	127N
1-15	PeV-A3 152037	133C	2-15	PeV-A3 152037	133C	3-15	PeV-A3 152037	133C
1-16	PeV-A3 178608	129C	2-16	PeV-A3 178608	129C	3-16	PeV-A3 178608	129C
1-18	PeV-A3 178608	133N	2-18	PeV-A3 178608	133N	3-18	PeV-A3 178608	133N
1-19	PeV-A3 51903	130C	2-19	PeV-A3 51903	130C	3-19	PeV-A3 51903	130C
1-21	PeV-A3 51903	132C	2-21	PeV-A3 51903	132C	3-21	PeV-A3 51903	132C
1-25	E11 50473	127C	2-25	E11 50473	127C	3-25	E11 50473	127C
1-26	E11 50473	131N	2-26	E11 50473	131N	3-26	E11 50473	131N
1-29	MOCK	129N	2-29	MOCK	129N	3-29	MOCK	129N
1-30	MOCK	128C	2-30	MOCK	128C	3-30	MOCK	128C
Linker	Pool of 16 samples	134C	Linker	Pool of 16 samples	134C	Linker	Pool of 16 samples	134C
