# Supplementary material for: Parechovirus infection in human brain organoids: host innate inflammatory response and not neuro-infectivity correlates to neurologic disease
Source: Nat Commun. 2024 Mar 21;15:2532. doi: 10.1038/s41467-024-46634-9 (PMC10958052; doi:10.1038/s41467-024-46634-9)
Supplement: Supplementary file 3 — Description of Additional Supplementary Files [file 41467_2024_46634_MOESM3_ESM.pdf]

Description of additional supplementary files

Title: Supplementary Data 1.

Description: Allocation of specific DAPs represented in Venn diagram (Figure 3c).

Title: Supplementary Data 2.

Description: Allocation of specific DAPs represented in Venn diagram (Figure 5c).

Title: Supplementary Movie 1.

Description: Representative video of a UNO section (20 μm). Note the astrocyte-rich areas (GFAP, red) and neuron-rich areas (MAP2, magenta) located outside the ventricular zones, high in neural progenitor cells (SOX2, yellow).

Title: Supplementary Movie 2.

Description: Representative video of PeV-A1 infected UNOs and stained for nuclei (cyan), and immunolabelled for PeV-A1 VP1 (magenta) and dsRNA (yellow).

Title: Supplementary Movie 3.

Description: Representative video of PeV-A3 infected UNOs and stained for nuclei (cyan), and immunolabelled for PeV-A3 VP3 (magenta) and dsRNA (yellow).
