# Supplementary material for: Parechovirus infection in human brain organoids: host innate inflammatory response and not neuro-infectivity correlates to neurologic disease
Source: Nat Commun. 2024 Mar 21;15:2532. doi: 10.1038/s41467-024-46634-9 (PMC10958052; doi:10.1038/s41467-024-46634-9)
Supplement: Supplementary file 4 — Supplementary Data 1 [file 41467_2024_46634_MOESM4_ESM.pdf]

[PeV-A1 Harris]: CDC42EP4,PEA15,VGF,CTSB,PSAT1,IDH2,TPI1,CNN3,DDAH1,APMAP,MAP4,QKI,DAP,ATP1A3,PPP1R1B,AHNAK,INPPL1,NES,UBE2L5,UQCRC1,PHB2,UQCRC2,PSMB6,YBX1,PGK1,GSN,AKT1S1,ECI2,PDLIM3,C11orf68,NAXE,CCT5,ATP6V1C1,ALDH6A1,H2AC4,ENSA,ATP6V1A,SCAMP1,ACO1,PARK7,PALD1,HTRA1,PLEC,LLGL1,CBR1,DDX42,DBNL,NUCB1,PGAM5,CHMP3,ATL1,PRKCSH,AKR1C2,ALDH9A1,FLNA,GATAD2A,TUBB3,MAPK1,TNKS1BP1,PRDX4,SYP,C8orf33,RO60,RUVBL1,STX1B,HSPA13,ATG3,ACADVL,NOL3,CCT8,RAPH1,L1CAM,UQCRFS1P1,SCRN1,C1QTNF4,ERCC1,GOT2,PSMD1,GNB1,USP5,CHCHD6,TPP1,MPI,RPN1,ELAC2,DLST,PPIA,NUTF2,DCTN1,SOGA3,S100B,HADHA,RAB3A,STOML2,PLPPR3,PGRMC1,GNAI3,MVB12B,PALLD,OPTN,SEPTIN5,TCP1,GMFB,GPRIN1,NDRG2,GYS1,ZFR,LRRC40,HMOX2,ENOPH1,PDCL,PDLIM5,CSRP2,C11orf54,SCG2,RBMX,ITPKB,HOPX,YARS1,RCN2,SERBP1,TSSC4,EDIL3,MSN,ATP5PF,WNT2B,CASP6,PHGDH,HYOU1,ATP1B1,ADD3,SEZ6L,WDR91,FASN,CHGB,SST,PPP2CA,NEDD8,HSPH1,STMN4,PMVK,TMED10,CXADR,ADPRS,PSMA3,POLR3GL,SEC24C,ACSL4,TFAM,CHTOP,NEBL,LTA4H,PRRT2,CCDC6,PIP4K2B,CRYZ,HSPB1,SH3PXD2B,LONP1,PFKL,MYO18A,NDUFS3,PDIA4,SEC61B,CMPK1,UGGT1,PTS,NDUFC2,ATP6V1B2,PEPD,SF3B2,MYH10,CDS2,HSPA1B,DAG1,TMED4,VAPB,ZNF768,GAPDHS,HSDL2,H2AZ1,STXBP1,PCSK1,GSTP1,DPYSL5,EIF4G1,ACLY,ATP6V0A1,CACNA2D1,MOB4,CKMT1A,EEF1D,CCT3,TPPP3,AIMP1,MYO5A,DDAH2,EIF3K,MPRIP,HK1,ABLIM1,NUDCD2,IMMT,NAPB,AK2,NAPA,PSMD7,NEGR1,GLO1,HMGN1,KIF5C,ALDH16A1,ELAVL3,PLIN3,COPS3,NMT1,HMGB2,OPA1,PDIA6,TMPO,CCNY,WFS1,HECA,RBM26,WASHC3,AK1,IK,KHSRP,WNK1,PSMD3,SPAG9,PRSS22,GNG3,MTMR8,RANBP6,ATP5IF1,ADGRG1,SSR1,CPD,NFKB1,ST13,GRHPR,CDKN2D,PLOD3,TMOD2,NCAM1,TEX264,RANBP2,TJP1,TMEM30A,SACM1L,TM9SF3,BLOC1S4,PLEKHA5,LSAMP,NAV1,CTSD,HIKESHI,DIABLO,SSR4,SELENBP1,CKM,SLC25A3,HMGN5,PGM1,STXBP3,AKT3,CYC1,OGFR

[PeV-A3 152037]: EIF2AK2,IFI44L,LAP3,STAT1

[E11]: PRPH,CD44,NAMPT,PDLIM1,WLS,STOM,LGALS3,GNG12,GPSM1,SLC25A11,FAM162A,AGPAT1,PEF1,TXLNA,DNAJB11,RRM1,MCAM,COL3A1,TNC,RP2,SEC23A,RPS14,SRSF1,PITHD1,FSD1,ASF1A,SRP72,GATD3B,GFAP,SYNM,SNRPD3,GRPEL1,H2BC26,FLOT1,SNCA,ATXN3,COPS5,ATXN10,SORBS1,ACOT2,APIP,LGALSL,RPL30,ATP5F1C,STAT3,PSMB1,SRSF9,RAD50,PASK,PTGIS,PIP4P1,FERMT2,EIF3E,COL1A1,UBE2I,RAB1A,RPS24,RRAGC,TRIM25,CRABP2,CRK,SNX6,SEC24A,PHF5A,TOMM70,TCERG1,GLUL,RAB3C,DOCK5,HNRNPL,SRSF3,ELOC,CNPY4,WDR47,ENY2,GNS,GMPS,NAP1L1,GATM,ACOT13,DHX36,CBX1,STARD10,PSMD10

[PeV-A1 Harris] and [E11]: GANAB,CCDC30,CNTFR,FH,FKBP1A,POM121,SUGP2,RPL9,TNRC6B,GLG1,AKR1A1,CHCHD3,PSMB2,CLU,GOLIM4,METAP2,FKBP3,SSBP1,CUL1,SLIRP,SLC25A22,UBE2N,LRPAP1,NGFR,PDK1,HSPD1,ESD

[PeV-A1 Harris] and [PeV-A3 152037] and [E11]: LASP1,CCDC50

[PeV-A1 Harris] and [PeV-A3 152037]: ISG15,IFIT2,EDF1,OAS3,MX1,IFIT3
