# Supplementary material for: Parechovirus infection in human brain organoids: host innate inflammatory response and not neuro-infectivity correlates to neurologic disease
Source: Nat Commun. 2024 Mar 21;15:2532. doi: 10.1038/s41467-024-46634-9 (PMC10958052; doi:10.1038/s41467-024-46634-9)
Supplement: Supplementary file 5 — Supplementary Data 2 [file 41467_2024_46634_MOESM5_ESM.pdf]

[clinical PeV-A1]: NAXE,IQGAP2,SCN2A,Q9H1Z4,RUFY3,ATP5IF1,AK3,TBC1D24,RAD21,NPEPL1,COPG2,PYCR1,THRAP3,MRI1,ATP5MJ,SLC27A1,SRSF2,HSDL1,TMUB1,PCK2,SENP8,FBXO45,FAM98B,APEH,GATAD2A,NFS1,ELAC2,PPAT,DHRS11,PTPRA,PALD1,PRPF31,WNK2,ACTC1,SDHB,ZNG1F,PDCD5,TTR,HEXB,EIF4E,NFKB1,UBXN7,TBC1D17,TIMM9,CAPS,PIP4P2,NACC1,REXO2,UBXN4,ABCE1,SLC39A10,SF3B1,IDE,ADPRS,ZNF768,BRSK1,EPHX1,DCAKD,COL4A2,GDPD2,ABCB6,WNT2B,TBC1D10B,EIF3I,SCAMP3,DDX39A,ATP6AP1,SNRPG,SEC61B,CPT1C,TIMP2,UTP18,NOTCH2,TMEM132A,EIF2S1,MAP2K2,ACLY,UQCRC1,POP1,CCDC6,PRKCI,PCYOX1L,AKT3,RAB1B,CTNNBL1,UROS,CYTH2,NIBAN2,CYC1,PACS1,MTA2,STIP1,PPP1R9B,TFE3,AK1,EDIL3,EHMT1,WASF1,CCDC88A,CAPZB,IWS1,LIN28A,SRRT,GRAMD1A,BOLA2,XIRP2,OAF,GMFB,ABHD16A,UBE2M,HSPA1L,PITRM1,METTL26,SUCLG2,VPS36,DGKE,WASF3,IRGQ,HIP1,PDE6D,SERPINB6,MLF2,RO60,VARS1,PSEN1,FKBP2,UBE2V2,SAT2,URI1,SOD2,TUBA4A,TSPAN18,ARL8B,ACAA1,RRAGB,AKT1,H2BC5,PHRF1,TOLLIP,EXOC3,ANKMY2,COMT,ACSL4,CUTC,PARD6B,PPP1CC,NHLRC2,TUBB,GSK3A,GLB1,CSTB,IK,CSTF3,AMPD2,MAPRE2,C8orf33,STXBP6,AKAP8,STK24,MTUS2,HS2ST1,MEST,FXYD7,PPP5C,EML2,EIF3C,MAP2K7,LRRFIP2,FAM114A1,CYFIP1,PPP1R9A,STXBP3,ABI1,PRDX4,ARHGAP17,STX4,NOP2,LRRC40,BCAP29,C12orf4,THSD7A,ARMCX1,NDUFS2,RAN,CST3,IMPDH2,PAXX,EI24,WDR37,SACM1L,LENG8,UBAP2L,BTRC,CNPY2,PABPN1,ACAP1,UBE2O,ROBO1,GLT8D1,LAMTOR4,DNAJC11,CLTA,DHRS4,ENOPH1,KDSR,DCC,SUGT1,M6PR,UROD,NPPA,ITGA5,USP10,AP3S1,GPS1,GLE1,HNRNPD,LSM7,DCTN1,C19orf53,VPS35,CDC26,SCARB2,IMMT,ELP2,H2BC18,MPPED1,OLA1,TMSB10,TYRP1,TSC1,STAM,DDX27,CUL4B,HECA,GTF2F1,EIF3K,SEZ6L,PCYT1A,SCAMP1,COL6A3,ITGA6,RPL18,EIF3A,DFFA,PHF14,ELP1,COL23A1,TNPO2,RRAS2,SNRNP40,GFM1,FDXR,FAM169A,ANGPTL4,ALG1,LSAMP,SDF2L1,SEL1L,DLAT,SH3BGRL,THOP1,ATP5MF,RBM10,ADSS2,FECH,EIF4EBP2,IGFBP7,GOSR1,SNX5,KIF3C,SEPTIN7,PITPNM1,ZNF330,SDHA,TRIM56,FYN,PLP1,BCAN,SAMHD1,RPA3,RPL35,ZNRD2,UBE4A,SEPSECS,PPARA,GNAI1,PALM

[Clinical PeV-A3]: IFIT2,PSMA6,EMD,ISG15,HP1BP3,SET,RPL24,MX1,PSMA3,TPP2,ARGLU1,PHB1,AOC1,PSMD1,PRNP,H1-0,PLIN3,NEBL,DLD,TOMM20,MAP4,HNRNPA0,GMPR2,BPNT1,DNAAF5,CHD4,C11orf54,PSMA1,FHL1,RALYL,OAS3,SYNJ1,PSMB3,IMP3,EIF3D,H2BC3,COX5B,SLC1A5,IFI44L,PPCS,CUEDC1,PSMB5,STOML2,TRH,APP,NES,MIF,MACROH2A2,WNK1,NBDY,RUVBL2,TPD52L1,RECQL,CFL1,RNH1,ZNF780B,IFIT3,OGDH,CCT5,NTN4,CMAS,LAMTOR1,GLO1,PGM1,HMGA1,EIF4B,PSMD6,PSMA5,GRSF1,MRPL50,CLIP1,MAPK3,NARS1,MCCC2,MRPL15,CHMP4B,SCP2,HOPX,TP53BP1,PGK1,AHNAK,CHTOP,HSPA5,GAN,IFIT1,WIPI2,NOL3,MAP3K2,TPR,DSC2,PGK2,LZTFL1,DUSP3,PRPS2,NDUFV1,MPRIP,PDZD8,CD99,GAA,CDK11B,BLVRB,PNO1,ATXN2L,NCK2,RPL7A,ECH1,INPPL1,PLCH1,MGMT,ARPC5,TCEAL5,PAICS,MACROH2A1,NKTR,ATP6V1E1,RAB11B,AP1G1,GABARAP,DAG1,PMPCB,NUP88,TUBA1C,CROCC,HMGN5,ISOC1,GCLC,MPI,FAHD2A,ALDH9A1,MFAP1,LUC7L,LTBP1,WASHC4,FTH1,PREB,ENSA,ATXN2,GAMT,PRAF2,TCP1,MARCKS,NOP58,B3GAT3,ADD3,C6orf89,CHMP1A,MYDGF,AQR,PICALM,CTTNBP2NL,ALPL,APBB1,MESD,CSNK1D,UBTF,TECR,PLCXD3,TTN,PALS2,LSS,HMOX2,ATP5F1D,ERP44,PPIL3,ARPC4,AAR2,DNAJB2,ARID2,LMNB1,COPS7B,CARHSP1

[E11]: CD44,WLS,SSBP1,LGALS3,GNG12,GPSM1,SLC25A11,POM121,AGPAT1,PEF1,DNAJB11,RRM1,MCAM,COL3A1,ESD,TNC,CLU,SEC23A,RPS14,SRSF1,PITHD1,PDK1,FSD1,ASF1A,SRP72,GATD3B,GFAP,SYNM,SNRPD3,H2BC26,GOLIM4,SNCA,UBE2N,FKBP1A,ATXN3,COPS5,SORBS1,ACOT2,APIP,LGALSL,RPL30,ATP5F1C,STAT3,NGFR,RAD50,PASK,PTGIS,PIP4P1,FERMT2,EIF3E,UBE2I,RAB1A,GLG1,RPS24,RRAGC,TRIM25,CRABP2,CRK,SNX6,SEC24A,PHF5A,TOMM70,TCERG1,GLUL,RAB3C,DOCK5,HNRNPL,FH,ELOC,WDR47,ENY2,FKBP3,GMPS,NAP1L1,GATM,ACOT13,SLIRP,DHX36,CBX1,STARD10

[clinical PeV-A1] and [E11]: CUL1,SRSF9,RPL9,LRPAP1,ATXN10,GNS,CNPY4,FLOT1,RP2,TNRC6B,PSMB2,AKR1A1,CCDC30,COL1A1,TXLNA,METAP2,SLC25A22,SRSF3,STOM,PDLIM1,NAMPT

[Clinical PeV-A3] and [E11]: FAM162A,PSMD10

[clinical PeV-A1] and [Clinical PeV-A3] and [E11]: PSMB1,HSPD1,SUGP2,CHCHD3,CCDC50,GANAB,GRPEL1,LASP1,PRPH,CNTFR

[clinical PeV-A1] and [Clinical PeV-A3]: ZNF428,OPTN,CDC42EP4,PCCB,SEC24C,SUCLG1,USP5,FAM133A,RUVBL1,SNRNP70,ALDH16A1,ARSA,HTRA1,RTCA,ARCN1,HSPA1B,ADAMTS3,PEX5,AGO1,HEXA,SFPQ,MEAK7,MED22,CCT3,NR2C2AP,FKBP15,RPL5,CASP6,NUCB1,MAGEA8,PRKCSH,OSGEP,TPI1,PMVK,TXNDC12,UFD1,PFAS,SEC11A,NAE1,ATP6V1B2,ACADS,AIP,ART4,PSAT1,CLNS1A,CCT6A,MAN2C1,TRIM2,FAHD1,TMX4,GNB4,ABCF3,SNX3,CTNNA2,CNPY3,USP15,GYS1,OARD1,ACP2,VGF,HAUS1,CHMP1B,PPP1R11,METAP1,PDZD11,PRDX3,CTSB,ZMYND8,ELOB,SLC16A3,EMILIN3,PPP1R1B,SOGA3,GPI,PCCA,PGAM5,EXOSC6,EDF1,LEO1,ANP32B,LONP1,TOMM6,MT-CO2,NXN,NDUFS3,PSMB6,CHMP4A,VIM,PNPT1,MICAL3,HRG,SMOC1
