# Supplementary material for: Parechovirus infection in human brain organoids: host innate inflammatory response and not neuro-infectivity correlates to neurologic disease
Source: Nat Commun. 2024 Mar 21;15:2532. doi: 10.1038/s41467-024-46634-9 (PMC10958052; doi:10.1038/s41467-024-46634-9)
Supplement: Supplementary file 9 — Reporting Summary [file 41467_2024_46634_MOESM9_ESM.pdf]

Corresponding author(s): Katja C. Wolthers

Last updated by author(s): Feb 21, 2024

Reporting Summary

Nature Portfolio wishes to improve the reproducibility of the work that we publish. This form provides structure for consistency and transparency in reporting. For further information on Nature Portfolio policies, see our Editorial Policies and the Editorial Policy Checklist.

Statistics

For all statistical analyses, confirm that the following items are present in the figure legend, table legend, main text, or Methods section.

n/a	Confirmed	
☐	☒	The exact sample size (*n*) for each experimental group/condition, given as a discrete number and unit of measurement
☐	☒	A statement on whether measurements were taken from distinct samples or whether the same sample was measured repeatedly
☐	☒	The statistical test(s) used AND whether they are one- or two-sided<br>*Only common tests should be described solely by name; describe more complex techniques in the Methods section.*
☒	☐	A description of all covariates tested
☐	☒	A description of any assumptions or corrections, such as tests of normality and adjustment for multiple comparisons
☐	☒	A full description of the statistical parameters including central tendency (e.g. means) or other basic estimates (e.g. regression coefficient) AND variation (e.g. standard deviation) or associated estimates of uncertainty (e.g. confidence intervals)
☐	☒	For null hypothesis testing, the test statistic (e.g. *F*, *t*, *r*) with confidence intervals, effect sizes, degrees of freedom and *P* value noted<br>*Give P values as exact values whenever suitable.*
☒	☐	For Bayesian analysis, information on the choice of priors and Markov chain Monte Carlo settings
☒	☐	For hierarchical and complex designs, identification of the appropriate level for tests and full reporting of outcomes
☒	☐	Estimates of effect sizes (e.g. Cohen's *d*, Pearson's *r*), indicating how they were calculated

Our web collection on statistics for biologists contains articles on many of the points above.

Software and code

Policy information about availability of computer code

Data collection	Leica LAS AF Software (Leica Microsystems), LAS-X 3D software (Leica Microsystems), CFX Maestro 1.1, Bio-Plex Manager Software v6.2, Proteome Discoverer 3.0
Data analysis	GraphPad Prism 8 (GraphPad Software Inc), NormFinder Excel Add-In (*.xla, MS Excel 2003) v 0.953, ImageJ 1.50I, R package NormalyzerDE v1.12.0, R package impute v1.68.0, R package sva v3.42.0, R package Linear Models for Microarray Data (limma) v3.50.0, R package PIANO v2.10.0., Interactivenn, Adobe Illustrator 2023, package ggplot2 v3.4.2, R package ComplexHeatmap v2.10.0.

For manuscripts utilizing custom algorithms or software that are central to the research but not yet described in published literature, software must be made available to editors and reviewers. We strongly encourage code deposition in a community repository (e.g. GitHub). See the Nature Portfolio guidelines for submitting code & software for further information.

Data

Policy information about availability of data

All manuscripts must include a data availability statement. This statement should provide the following information, where applicable:

- Accession codes, unique identifiers, or web links for publicly available datasets
- A description of any restrictions on data availability
- For clinical datasets or third party data, please ensure that the statement adheres to our policy

The raw data supporting the conclusions of this article is uploaded in figshare.com and will be public once the manuscript is published. Sequencing results will be available on GenBank (accession numbers BankIt: E11 OR886062 and PeV-A OR886056-OR886061). The mass spectrometry proteomics data have been deposited

to the ProteomeXchange Consortium via the PRIDE partner repository with the dataset identifier PXD047238

Used Databases:
Homo sapiens consensus protein database (UniProt TaxID=9606 v2023-02-09) - https://www.uniprot.org/taxonomy/9606
human Molecular Signatures Database (MSigDB) - https://doi.org:10.1016/j.cels.2015.12.004

Research involving human participants, their data, or biological material

Policy information about studies with human participants or human data. See also policy information about sex, gender (identity/presentation), and sexual orientation and race, ethnicity and racism.

Reporting on sex and gender	No human research participants were used for this study.
Reporting on race, ethnicity, or other socially relevant groupings	No human research participants were used for this study.
Population characteristics	No human research participants were used for this study.
Recruitment	No human research participants were used for this study.
Ethics oversight	No human research participants were used for this study.

Note that full information on the approval of the study protocol must also be provided in the manuscript.

Field-specific reporting

Please select the one below that is the best fit for your research. If you are not sure, read the appropriate sections before making your selection.

☒ Life sciences ☐ Behavioural & social sciences ☐ Ecological, evolutionary & environmental sciences

For a reference copy of the document with all sections, see nature.com/documents/nr-reporting-summary-flat.pdf

Life sciences study design

All studies must disclose on these points even when the disclosure is negative.

Sample size	No sample size calculation was performed. Brain organoids of three independent differentiations were used and each of the three batches infected in an independent experiment with a sample size of 3 organoids per experiment. The aim for this sample size was to have a sufficient number of samples to keep the chance of errors at an acceptable level while avoiding to make the study unreasonable large, taking time, costs and necessary material into consideration.
Data exclusions	Datapoints from Figure 1b and c, Figure 3a and b, and Figure 4 were excluded based on a outliers test (ROUT Q= 1%) that was performed on the raw data sets. Data from figure 4 was excluded when a majority of the datapoints were below the limit of detection. In case of a minority of values that were below the lower limit of detection (LLOD) within a condition, these values were replaced by the LLOD/√2. For more details on the LLOD/√2 method see reference: Croghan, C. W., Egeghy, P.P. Methods of Dealing with Values Below the Limit of Detection using SAS, <http://analytics.ncsu.edu/sesug/2003/SD08-Croghan.pdf (> (2003).
Replication	To ensure reproducibility the generation and infection of brain organoids was performed three times independently. The data used in the paper are from those three experiments and no extra attempts at replication were performed. We did not observe any findings that suggested that this experiment cannot be reproduced.
Randomization	Samples (brain organoids) were randomly allocated to the different conditions analyzed in this study. No pre-selection of brain organoids was performed in order to generate specific outcomes of this study.
Blinding	During data analysis and collection no measures were taken to obtain blinding. Blinding was not relevant to our study as performance bias was not considered a risk in our experimental setup.

Reporting for specific materials, systems and methods

We require information from authors about some types of materials, experimental systems and methods used in many studies. Here, indicate whether each material, system or method listed is relevant to your study. If you are not sure if a list item applies to your research, read the appropriate section before selecting a response.

Materials & experimental systems

n/a	Involved in the study
	[x] Antibodies
	[x] Eukaryotic cell lines
[x]	Palaeontology and archaeology
[x]	Animals and other organisms
[x]	Clinical data
[x]	Dual use research of concern
[x]	Plants

Methods

n/a	Involved in the study
[x]	ChIP-seq
[x]	Flow cytometry
[x]	MRI-based neuroimaging

Antibodies

Antibodies used

Antigen (abbreviation), Host, Dilution factor, Company, Catalogue #
- Special AT-rich sequence-binding protein 2 (SATB2) Mouse 1:400 Abcam Ab51502
- Chicken ovalbumin upstream promoter transcription factor-interacting protein 1 (CTIP2) Rat 1:500 Abcam Ab18465
- Paired box protein 6 (PAX6) Rabbit 1:300 Stemcell Technologies STC60094
- Glial fibrillary acidic protein (GFAP) Goat 1:500 Abcam Ab53554
- Sex determining region Y-box 2 (SOX2) Rabbit 1:400 Cell Signaling Technologies 34516
- Microtubule-associated protein 2 (MAP2) Mouse 1:500 Thermo Fisher MA5-12826
- dsRNA clone rJ2 Mouse 1:60 Sigma-Aldrich MABE1134
- Parechovirus 1 VP1, rabbit, 1:300, Thermo Fisher, custom made
- Parechovirus 3 VP1, rabbit, 1:300, Thermo Fisher, custom made

Secondary Antibody Host Dilution factor Company Catalogue #
- Anti-mouse Alexa Fluor 546 Donkey 1:750 Thermo Fisher A10036
- Anti-rat Alexa Fluor 488 Donkey 1:750 Thermo Fisher A48268
- Anti-rabbit Alexa Fluor 680 Donkey 1:750 Abcam Ab175772
- Anti-goat Alexa Fluor 594 Donkey 1:750 Thermo Fisher A11058

Validation

The primary antibodies were validated in our lab using positive control samples. In the experimental analysis of ICC samples a negative control lacking primary antibodies was taken along to control for the non-specific antibody binding to the sample. The dilution used was based on recommendations on manufactures websites and previously used dilutions by fellow scientist reported on Citeab.

- Special AT-rich sequence-binding protein 2 (SATB2) Mouse 1:400 Abcam Ab51502: According to Abcam suitable for ICC, reacts with human and recommended to use a concentration of 2 - 100 µg/ml. Citeab recommends dilution of 1:50-1:500 when using for ICC.
https://www.abcam.com/satb1--satb2-antibody-satba4b10-c-terminal-ab51502.html
https://www.citeab.com/antibodies/772164-ab51502-anti-satb1-satb2-antibody-satba4b10-c-t

- Chicken ovalbumin upstream promoter transcription factor-interacting protein 1 (CTIP2) Rat 1:500 Abcam Ab18465: According to Abcam suitable for ICC and IF and reacts with human samples. Recommended dilution is 1:500.
https://www.abcam.com/ctip2-antibody-25b6-ab18465.html

- Paired box protein 6 (PAX6) Rabbit 1:300 Stemcell Technologies STC60094: According to Stemcell Techonologies to react with PAX6 of humans and other species, is verified and reported to be suitable for ICC with a suggested dilution between 1:300-1:500.
https://cdn.stemcell.com/media/files/pis/27685-PIS_1_1_0.pdf

- Glial fibrillary acidic protein (GFAP) Goat 1:500 Abcam Ab53554: According to Abcam will react with human GFAP. According to Citeab previously used for ICC using dilutions 1:1000 and 1:200.
https://www.citeab.com/antibodies/732883-ab53554-anti-gfap-antibody
https://www.abcam.com/gfap-antibody-ab53554.html

- Sex determining region Y-box 2 (SOX2) Rabbit 1:400 Cell Signaling Technologies 34516: According to Cell Signaling Technologies reacts with human SOX2 and can be used for ICC as an application at a recommended dilution of 1:400
https://www.cellsignal.com/products/primary-antibodies/sox2-d6d9-xp-rabbit-mab/3579

- Microtubule-associated protein 2 (MAP2) Mouse 1:500 Thermo Fisher MA5-12826: According to thermofisher reacts with MAP2 in human neurons derived from iPSCs and can be used at a dilution of 1:1000
https://www.thermofisher.com/nl/en/home/technical-resources/research-tools/image-gallery/image-gallery-detail.110719.html

- dsRNA clone rJ2 Mouse 1:60 Sigma-Aldrich MABE1134: According to Merck, can react with double stranded RNA of viral species and has been tested for use in ICC an IF. Dilutions recommended is 1:60.

Eukaryotic cell lines

Policy information about cell lines and Sex and Gender in Research

Cell line source(s)	human induced pluripotent cell line IMR90-4 commercially available at WiCell.<br>Cell line Alias: iPS(IMR90) clone (#4),<br>hPSCReg ID: WISCi004-B<br>Sex: Female<br><br>References:<br>- Induced Pluripotent Stem Cell Lines Derived from Human Somatic Cells- Junying Yu,1,2* Maxim A. Vodyanik,2 Kim Smuga-Otto,1,2 Jessica Antosiewicz-Bourget,1,2 Jennifer L. Frane,1 Shulan Tian,3 Jeff Nie,3 Gudrun A. Jonsdottir,3 Victor Ruotti,3Ron Stewart,3 Igor I. Slukvin,2,4 James A. Thomson1,2,5*<br>- https://www.wicell.org/home/stem-cells/catalog-of-stem-cell-lines/ips-imr90-4.cmsx<br><br>Immortalized Cell lines<br>Vero - African green monkey kidney cells (Vero, provided by the National Institute of Public Health and the Environment, RIVM, the Netherlands)<br>LLCMK2- rhesus monkey kidney cells (LLCMK2, provided by the Municipal Health Services, the Netherlands)<br>HT-29- Human colorectal adenocarcinoma cells (HT-29, ATTC HTB-38)
Authentication	Identification of the immortalized cell lines is confirmed on a regular basis as part of quality control system in our laboratory by morphology. With the use of an external party (IDEXX BioAnalytics Europe) we confirm the identity of the cells by STR profiling using the test CellMark TM.
Mycoplasma contamination	All cell lines were repeatedly tested for mycoplasma contamination when generating the cell bank and before use in the experiment using MycoAlert Mycoplasma Detection Kit (Lonza). In addition obtained samples at the end of the experiments were tested. All tests came out negative for mycoplasma contamination.
Commonly misidentified lines<br>(See ICLAC register)	No commonly misidentified lines stated in the ICLAC register v12 were used in our study

Plants

Seed stocks	n/a
Novel plant genotypes	n/a
Authentication	n/a
